# Effect of Polymer Network Architecture on Adsorption Kinetics at Liquid–Liquid Interfaces: A Comparison Between Poly(NIPAM-co-AA) Copolymer Microgels and Interpenetrating Network Microgels

**DOI:** 10.3390/gels11010058

**Published:** 2025-01-11

**Authors:** Galina A. Komarova, Elena Yu. Kozhunova, Rustam A. Gumerov, Igor I. Potemkin, Irina R. Nasimova

**Affiliations:** Physics Department, Lomonosov Moscow State University, Leninskie Gory 1-2, 119991 Moscow, Russia; komarova@polly.phys.msu.ru (G.A.K.); kozhunova@polly.phys.msu.ru (E.Y.K.); gumerov@polly.phys.msu.ru (R.A.G.); igor@polly.phys.msu.ru (I.I.P.)

**Keywords:** polymer microgels, interpenetrating networks microgels, poly(N-isopropylacrylamide), polyacrylic acid, smart microgels, interface behavior, emulsions stabilization

## Abstract

Understanding the adsorption features of polymer microgels with different chemical compositions and structures is crucial in studying the mechanisms of respective emulsion stabilization. Specifically, the use of stimuli-responsive particles can introduce new properties and broaden the application range of such complex systems. Recently, we demonstrated that emulsions stabilized by microgels composed of interpenetrating networks (IPNs) of poly-N-isopropylacrylamide (PNIPAM) and polyacrylic acid (PAA) exhibit higher colloidal stability upon heating compared to PNIPAM homopolymer and other relevant PNIPAM-based copolymer counterparts. In the present work, using pendant drop tensiometry, we studied the evolution of water–tetradecane interfacial tension during the adsorption of PNIPAM-PAA IPN particles, comparing them with single-network P-(NIPAM-co-AA) and PNIPAM microgels. The results showed that, despite having the same chemical composition, copolymer particles exhibit completely different adsorption behavior in comparison to other microgel architectures. The observed disparity can be attributed to the nonuniform distribution of charged acrylic acid groups within the P-(NIPAM-co-AA) network obtained through precipitation polymerization. Oppositely, the presence of IPN architecture provides a uniform distribution of different monomers inside respective microgels. Additionally, hydrogen bonding between PNIPAM and PAA subchains appears to reduce the electrostatic energy barrier, enhancing the ability of IPN particles to successfully cover the liquid interface. Overall, our findings confirm the efficiency of using PNIPAM-PAA IPN microgels for the preparation of oil-in-water emulsions and their stability, even when the temperature rises above the lower critical solution temperature of PNIPAM.

## 1. Introduction

Polymer microgels, especially thermo- and pH-sensitive ones, have attracted significant attention from researchers over the past few decades. Microgels are soft, three-dimensional polymer colloidal particles consisting of a cross-linked network that is swollen in a good solvent (usually water) [1,2]. They can form stable dispersions and, like macrogels of the same nature, undergo a reversible volume phase transition from a swollen to a deswollen state in response to changes in their environment [3,4,5,6,7]. These properties determine their potential applications, including superabsorbents, surface-active stabilizing additives, carriers for drug delivery, etc. [8,9,10,11,12].

Recently, another application for polymer microgels has been demonstrated: they can serve as stabilizing agents in emulsion formation. It has been shown that microgels can spontaneously adsorb to water–oil interfaces, thereby lowering the interfacial tension, which leads to the stabilization of emulsions [13,14,15,16]. Due to their high deformability, microgels tend to flatten out on the surface when adsorbed at the interfaces [17,18,19]. This distinguishes them from cases of emulsion stabilization by solid colloidal particles through the Pickering mechanism [20,21]. The properties of the emulsion primarily depend on the nature and fraction of the functional groups, their distribution, and, finally, the network architecture. The use of stimuli-responsive polymers enables the tuning of microgels’ swelling ratios through external stimuli such as temperature, pH, or solvent quality [3,22,23]. As a result, ‘smart’ emulsions are obtained—emulsions that can be broken in a controlled manner [24,25]. Such a unique feature can be beneficial in industrial applications, including interfacial catalysis [26,27], fuel production, and the collection or transportation of oil and petroleum products [28]. Currently, the most extensively studied materials for emulsion stabilization are microgels based on the thermosensitive polymer N-isopropylacrylamide and its copolymers with acrylic acid (AA) or methacrylic acid (MAA) [29]. This is partly due to the convenient method of synthesizing microgels through thermal precipitation polymerization, which allows for the production of microparticles without the need for additional components, such as surfactants [30,31]. In the case of copolymers, the resulting particles exhibit both temperature- and pH-responsive properties [32,33]. That is, the stability of the resulting emulsions can be controlled by altering both the temperature and the acidity of the solutions. For example, it has been demonstrated that emulsions stabilized by microgel particles based on a copolymer of NIPAM and methacrylic acid remain stable at low temperatures and high pH levels, but are destabilized at high temperatures and low pH [34].

As mentioned above, the behavior of particles at the phase boundary depends, among other factors, on the distribution of groups and the structure of the network. As demonstrated in several studies, microgels composed of copolymers of N-isopropylacrylamide with ionized monomer groups, obtained through precipitation polymerization, exhibit a nonuniform distribution of charged groups within their volume [35]. Most of the charged groups are located on the surface of the particles. This imposes restrictions on the selection of oil–water pairs and the fractions of liquid phases at which they could be stabilized.

A suitable solution for obtaining microgels with a uniform distribution of charged groups throughout their volume is to apply a step-wise polymerization process. This approach results in the formation of physically entangled interpenetrating networks that do not have any chemical bonds between them (IPN) [5,36,37,38]. In addition to facilitating the creation of microgels with a uniform distribution of charged units, the structure of interpenetrating networks provides the advantage that each network can respond independently—either by swelling or shrinking—to changes in external stimuli. This independence in the conformational behavior of the networks can lead to various intriguing effects. For example, it has been demonstrated that microphase separation can be observed at the scale of a single particle [39]. Regarding the liquid interface, one can expect that the independent response of two networks to external stimuli will expand the possibilities for stabilizing emulsions and tuning their properties. As far as we know, no one had studied this before our research on the emulsifying capacity of IPN microgels based on PNIPAM and PAA [40], nor has anyone compared it with other types of microgel particles of the same composition. In that study, we demonstrated that, in contrast to emulsions stabilized by homopolymer PNIPAM microgels, emulsions stabilized by PNIPAM-PAA IPN microgels can remain colloidally stable for an extended period, even after being heated to 40 °C, which exceeds the lower critical solution temperature (LCST) of PNIPAM. This stability was attributed to the presence of a charged polyacrylic acid network in microgels based on interpenetrating networks. However, comparisons with results obtained by other groups regarding the preparation of emulsions using microgels made from NIPAM copolymers with carbocyclic acids (for example, in refs. [24,41]) indicate that IPN microgels facilitate the formation of non-creaming emulsions that are homogeneous throughout the entire volume of the sample. To understand the stabilization mechanism of emulsions, mesoscopic computer simulations were performed to examine the conformational behavior of IPN microgels at the liquid-liquid interface. The simulations demonstrated that this behavior occurs because when the first subnetwork (PNIPAM) collapses, the particle adopts a flattened core–shell morphology, featuring a highly swollen PAA-rich shell and a collapsed PNIPAM-rich core. Thus, unlike the PNIPAM homopolymer and copolymer particles, the IPN microgel maintains its three-dimensional shape, providing stability to microgel-based emulsions over a wide range of temperatures. However, to better understand the features of emulsification by PNIPAM-PAA interpenetrating network (IPN) microgels, it is essential to study the dynamics of their adsorption at interphase boundaries, particularly in comparison with P-(NIPAM-co-AA) microgels. We have not previously published these data or their analysis. Therefore, in this paper, we will focus on measuring and analyzing the dynamic dependence of surface tension over time at the tetradecane-water interface for water solutions of three types of particles: homopolymer PNIPAM, P-(NIPAM-co-AA) copolymer microgels, and microgels based on PNIPAM and PAA interpenetrating networks. This issue is important because adsorption at the interface of two immiscible liquids plays a crucial role in the formation, stabilization, and breakdown of emulsions.

## 2. Results and Discussion

The kinetics of adsorption were investigated at temperatures of 20 °C and 40 °C. The choice of temperatures is based on the fact that PNIPAM is a thermo-sensitive polymer that undergoes a coil-globule transition at a lower critical solution temperature (LCST) of approximately 32 to 34 °C. At temperatures above LCST, water becomes a poor solvent for PNIPAM networks, causing PNIPAM microgels in bulk solution to adopt a collapsed state (as confirmed by the data on the change in particle radius presented in Table 1).

When microgels are adsorbed on the surface, they spread out over the surface, and a change in temperature also leads to a change in the conformation of PNIPAM microparticles. In the case of adsorption on the surface, according ref. [42], the structure of microgels at phase boundary is characterized by a core−corona morphology. The polymer segments within the interface lose their temperature sensitivity due to the strong adsorption at the interface, however, the part of the microgel particle that are located in the aqueous side of the interface maintains its temperature-sensitivity. Thus, temperature may influence the adsorption of microgels containing PNIPAM segments at interphase boundaries. Additionally, the charge and structure of the network are likely to affect adsorption.

**Figure 1:**
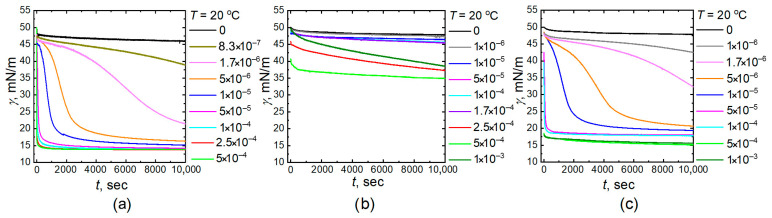
Influence of the microgel concentration on the dynamic interfacial tension (γ) as a function of time (*t*) at water–tetradecane interface at 20 °C ((**a**)—PNIPAM, (**b**)—P-(NIPAM-co-AA), (**c**)—PNIPAM-PAA IPN interfacial).

**Figure 2:**
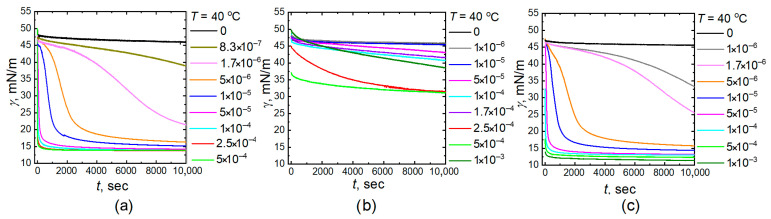
Influence of the microgel concentration on the dynamic interfacial tension (*γ*) as a function of time (*t*) at water–tetradecane interface at 40 °C ((**a**)—PNIPAM, (**b**)—P-(NIPAM-co-AA), (**c**)—PNIPAM-PAA IPN microgels).

Figure 1 and Figure 2 demonstrate the dynamic interfacial tension dependencies during the adsorption of all microgel particles (homo PNIPAM, P-(NIPAM-co-AA), and PNIPAM-PAA IPN) at the water-tetradecane interface at different concentrations. All plots demonstrate that the equilibrium final value of the interfacial tension decreases at 40 °C as compared to the tension at 20 °C. The equilibrium values of interfacial tension at 40 °C are approximately 3–4 mN/m less than at 20 °C for all NIPAM-based microgels. A decrease in the interfacial tension of pure liquids, as well as during the adsorption of low-molecular-weight surfactants with increasing temperature, is a common phenomenon. This decrease is associated with reduced cohesive forces—molecular adhesion in a liquid due to an increase in the average distance between molecules. It is interesting to note that for all investigated microgels with a temperature-sensitive component in their network structure, this difference is greater than that observed with low-molecular-weight surfactants, such as sodium oleate [43]. This indicates that by controlling the conformation of the polymer network, one can influence the adsorption capacity of particles and alter the surface tension. Indeed, the interfacial tension can be quantified by a number of unfavorable contacts of the molecules of two different liquids: the bigger the number of the contacts, the higher the tension. Therefore, adsorption of the microgel segments at the interface shields unfavorable liquid–liquid contacts reducing the interfacial tension: the higher the number of the adsorbed monomer units, the stronger the decrease in the interfacial tension. On the other hand, the adsorption results in the loss of conformational entropy of the microgel-forming subchains, and the magnitude of such energetic contribution depends on solvent quality. Specifically, at 20 °C, water acts as a good solvent for microgel segments, and thus, despite the deformations, the adsorbed particle remains strongly swollen in the water phase, which results in a less dense polymer layer [15,42,44,45,46]. In turn, at 40 °C water becomes a poor solvent, which, however, does not decrease the microgel deformation at the interface. Moreover, this results in a more flattened and dense conformation, providing a more effective screening of oil–water contacts since the loss of conformational entropy becomes less significant [42]. Additionally, microgel collapse provides an extra space at the interface for adsorption of additional particles from the bulk liquid. This explains the interfacial tension decrease upon heating.

Typically, the surface tension of the freshly formed interfacial surface of pure liquids or solutions of low-molecular-weight substances quickly reaches an equilibrium value. It can be observed from the presented surface tension dependences that the interfacial surface tension of the homo PNIPAM, P-(NIPAM-co-AA), and PNIPAM-PAA IPN microgel solutions at different concentrations decreases slowly over time, reaching an equilibrium value at low microgel concentrations. The rate of decrease is greatest in the initial stages, after which the decline slows down. It can also be noted that the time required to reach the equilibrium value decreases as the solution concentration increases. This behavior is typical for high-molecular compounds such as microgels. The decrease in interfacial tension over time is associated with the diffusion of surface-active substances from the bulk to the interphase boundary. In the case of low-molecular-weight surfactants, diffusion is not a limiting factor in the rate of establishing adsorption equilibrium [47]. The role of this factor cannot be excluded in the case of high molecular weight surfactants (for example, microgels), the large molecules have significantly lower mobility than the molecules (ions) of conventional amphiphilic surfactants.

**Figure 3:**
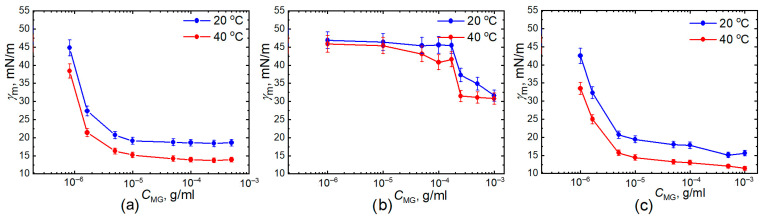
Meso-equilibrium interfacial tension *γ*_m_ as a function of microgel concentrations at 20 °C (blue) and 40 °C (red) for PNIPAM (**a**), P-(NIPAM-co-AA) (**b**), and PNIPAM-PAA IPN (**c**) microgel solutions. *γ*_m_ is the interfacial tension value at 10,000 s.

Figure 3 summarized the meso-equilibrium interfacial tensions (*γ*_m_ is the interfacial tension value at 10,000 s) as a function of microgel concentration at 20 °C and 40 °C for homo PNIPAM, P-(NIPAM-co-AA), and PNIPAM-PAA IPN microgels solutions. Similar trends are observed for PNIPAM (a) and PNIPAM-PAA IPN microgels (c): *γ*_m_ decreases significantly and then reaches a plateau for both temperatures. A distinct pattern of behavior is evident for microgels composed of P-(NIPAM-co-AA)copolymer (b). The surface tension decreases slightly at low concentrations, followed by a sharp drop, which may be attributed to the microgels overcoming the electrostatic barrier. In classical theory, adsorption kinetics is described using a model that assumes the presence of an energy barrier preventing the transition of surfactant molecules from the surface layer to the interphase surface [47]. This barrier is complex in nature. In the case of ionic surfactants, the adsorption of the initial ions imparts an electric charge to the surface, leading to the formation of an electrical double layer. To adsorb the remaining ions, it is necessary to overcome the forces of electrostatic repulsion, which increases the time required to reach the equilibrium value of surface tension. The influence of charge presence in the polymer network of microgels made from copolymers with carboxylic acids on the adsorption and the behavior of these microparticles at interphase boundaries has been discussed in several previous studies [45,48,49]. Similar to our findings, it was observed that for microgels of PNIPAM copolymers with acrylic acid, a critical concentration is reached at which the adsorption process begins [48]. The higher the charge of the polymer network, the slower the decrease in water-tetradecane interfacial tension occurs. Additionally, it should also be taken into account that as was shown by computer simulations [45,46], the interfacial area occupied by a single particle with high charge content was found to be significantly lower than the one in the case of neutral particle due to the higher swelling ratio perpendicularly to the liquid-liquid interface. As a result, monolayers of charged microgels need to be compressed further.

It is quite interesting that the dependences of the meso-equilibrium interfacial tensions on microgel concentration for IPN microgels (Figure 3c), which also contain charged groups of polyacrylic acid in their composition, do not differ significantly from those of neutral microgels made of the PNIPAM homopolymer (Figure 3a). The presence of a second PAA polymer network in IPN microgel matrix does not drastically affect the surface tension at the water–tetradecane boundary regardless of temperature (red and blue curves in Figure 3a,c). However, the change in the degree of swelling (radius of gyration) of the IPN microgel microparticles with an increase in temperature above the LCST of PNIPAM is significantly less than that of the homopolymer microgels (data presented in Table 1). At the same time, the apparent difference between the curves in Figure 3b,c clearly demonstrates the effect of microgel architecture on their interfacial behavior. Namely, for copolymer P-(NIPAM-co-AA) microgels the concentration of PAA units within the network increases from particle center to its periphery [50]. On the contrary, in this direction the concentration of cross-links decreases [51]. Therefore, the properties of such microgels with high PAA content (about 23–25%mol) essentially resemble the ones of spherical micelles with PAA-rich corona and dense PNIPAM-rich core. In this regard, the increase in temperature raises the core density while the corona remains significantly swollen due to electrostatic interactions. This is further supported by the negligible change in the radius of the copolymer microparticles with increasing temperature, compared to the PNIPAM homopolymer microgels and small difference in the average intensity of scattering light (Table 1). Eventually, it suppresses the fast covering of the oil–water interface by colloidal networks. Meanwhile, for IPN microgels the concentration of PAA and PNIPAM units within the particle is more homogeneous than in the previous case [39,40] while the total PAA content is similar (about 29%). In turn, the strong hydrogen bonding between PNIPAM and PAA subchains [52] (especially at higher temperatures) apparently lowers the electrostatic energy barrier allowing for more effective covering of the liquid interface. Finally, the increase in temperature increases the internal segment density inside the IPN particles [40] thereby decreasing the interfacial tension at the level of the single particle. Simultaneously, the presence of PAA subchains in the corona prevents particle aggregation, which increases the stability of respective oil-in-water emulsions.

**Figure 4:**
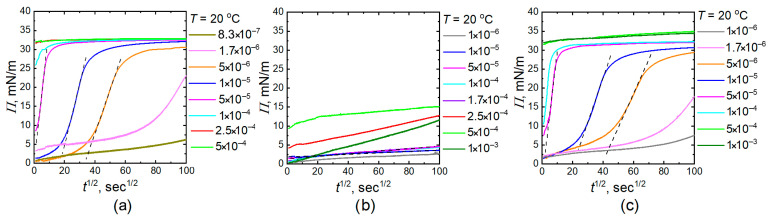
Surface pressure (*Π*) at water–tetradecane interface as a function of the square root of time for homo PNIPAM (**a**), P-(NIPAM-co-AA) (**b**), and PNIPAM-PAA IPN (**c**) microgels at 20 °C.

**Figure 5:**
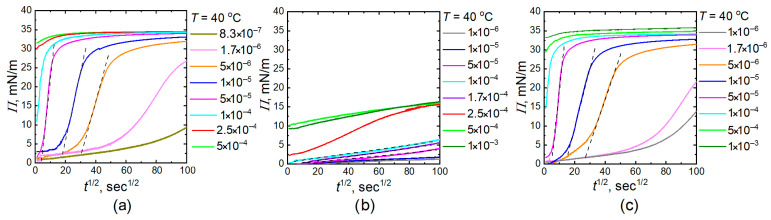
Surface pressure (*Π*) at water–tetradecane interface as a function of the square root of time for homo PNIPAM (**a**), P-(NIPAM-co-AA) (**b**), and PNIPAM-PAA IPN (**c**) microgels at 40 °C.

To facilitate the discussion of the differences in behavior between copolymer microgels and IPN microgels, we plotted curves that illustrate the relationship between surface pressure at the phase boundary and the square root of time for both 20 °C and 40 °C (Figure 4 and Figure 5). Surface pressure was calculated based on surface tension values according to the following formula:

(1) $$\Pi_{t}=\gamma_{0}-\gamma_{t}$$

where *γ*_0_ is the pristine interfacial tension equal to 50 mN/m (tetradecane/water interface).

It is interesting to note that, at lower concentrations of homo PNIPAM microgels and IPN microgels, the curve depicting the relationship between surface pressure and time takes on a sigmoid shape and can be considered as a steps process. Initially, there is a slow “induction” period, followed by a rapid increase in surface pressure, which is then succeeded by a slower rise until the curve reaches the equilibrium value.

For the average range of concentrations, the process of changing surface pressure occurs in two steps. The first step is marked by a sharp increase in surface pressure, followed by a gradual change that approaches equilibrium. Slow gradual change can be associated with the deformation and spreading of microgels at the interface, as well as the probable onset of microgel arrangement into a hexagonal packing, which occurs after they have filled the interphase. For microgels of PNIPAM copolymers with AA, the inflection is observed much later. For sufficiently high concentrations, the first step is too brief, and the surface tension almost immediately reaches its equilibrium value. At the concentration range where a rapid increase in surface tension was observed relatively quickly, we assumed that the first step corresponds to a purely diffusion-controlled process. According to the model presented in the ref. [53], the surface pressure is described by the following relation:

(2) $$\Pi_{t}=\gamma_{0}-\gamma_{t}=\Gamma_{t}RT=2RT\sqrt{\frac{D}{\pi }}C_{MG}\sqrt{t}$$

where *Π* is measured in mN/m and is proportional to the square root of time, with the slope *k_i_* depending on the concentration of microgels in the bulk. Thus, *Π* dependence on the square root of time were fitted linearly using equation:

(3) $$\Pi_{t}=k_{i}\sqrt{t}+C$$

where *C* is constant and the slope *k_i_* is:

(4) $$k_{i}=2RT\sqrt{\frac{D_{interfacial}}{\pi }}C_{MG}$$

**Figure 6:**
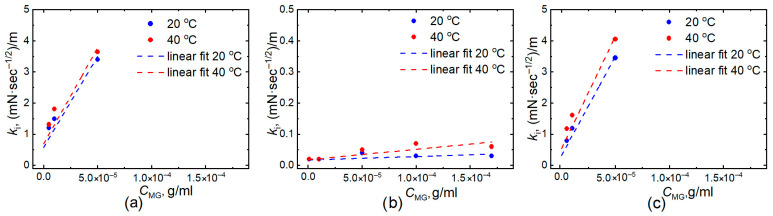
Plots of *k_i_* vs. microgel concentrations *C_MG_* at two temperatures: 20 °C (blue points) and 40 °C (red points) for homo PNIPAM (**a**), P-(NIPAM-co-AA) (**b**), and PNIPAM-PAA IPN (**c**) microgels.

The defined *k_i_* values plotted against the microgel concentration *C_MG_* in g/mL for all types of microgel particles at two temperatures (20 °C and 40 °C) are presented in Figure 6. In addition, using Equation (4), the interfacial diffusion coefficients (*D_interfacial_*) were determined. Then, *D_interfacial_* for PNIPAM and PNIPAM-PAA IPN microgels were normalized with *D_interfacial_* of P-(NIPAM-co-AA) microgels at 20 °C. The normalized values (*D^n^_interfacial_*) are presented in a Table 2. It is important to note that the table displays values normalized with respect to the *D_interfacial_* of P-(NIPAM-co-AA) microgels at 20 °C.

As can be seen, *kᵢ* is slightly higher at 40 °C for all types of microgels. For example, at a concentration value of 5∙10^−5^ g/mL, the parameter *k_i_* is 1.1/1.2/1.3 times greater at 40 °C compared to 20 °C for PNIPAM/PNIPAM-PAA IPN/P-(NIPAM-co-AA) microgels, correspondingly. However, when examining the normalized diffusion coefficients, *D^n^_interfacial_* for homopolymer PNIPAM at 20 °C is comparable to that at 40 °C. It can be assumed that the overall increase in diffusion associated with rising temperature is counterbalanced by the fact that the collapsed PNIPAM particles exhibit a less pronounced hydrophilic component and less effectively adsorbed at the oil boundary. When comparing different types of microgels, the diffusion coefficients of copolymer microgels are several orders of magnitude lower than those of PNIPAM homopolymer microgels and IPN microgels at both temperatures. The presented dependencies support the notion that the adsorption of charged copolymer microparticles onto the interphase boundary is challenging, resulting in lower surface pressure and a slower diffusion. Moreover, the diffusion coefficients for the IPN microgels are slightly higher than those for the PNIPAM microgels, which supports the higher emulsifying capacity of the IPN microgels that we demonstrated earlier [40].

## 3. Conclusions

In this work, we demonstrate the significant influence of the microgel network structure on their surface-active properties, based on data regarding the dependence of surface tension over time during the adsorption of microgel particles at the water-tetradecane interface. Despite the same chemical composition in terms of the ratio of monomer units of NIPAM and AA, charged copolymer microgels exhibit a completely different adsorption behavior compared to microgels based on interpenetrating networks of PNIPAM and PAA. Specifically, for copolymer microgels, there exists a critical concentration below which the adsorption of microgels onto the surface is challenging; this phenomenon is not observed in IPN microgels. Their surface activity closely resembles that of uncharged PNIPAM homopolymer microgels. The observed difference can be attributed to the structural features of the copolymer microgel network obtained through thermal precipitation polymerization used in this study. This network can be considered as spherical micelles with a PAA-rich corona and a dense PNIPAM-rich core. Consequently, all the characteristics of adsorption at the phase boundary of charged substances are evident in this system. Meanwhile, for IPN microgels, the concentration of PAA and PNIPAM units within the particle is more homogeneous, and the strong hydrogen bonding between PNIPAM and PAA subchains apparently lowers the electrostatic energy barrier allowing for more effective adsorption of the liquid interface. The obtained results confirm the high stabilizing ability of PNIPAM-PAA IPN microgel for preparing oil-in-water emulsions, which we previously demonstrated. Our findings highlight important factors in the study of emulsion stabilization by polymer microgels and their potential use in the preparation of ‘smart’ emulsions. Based on our results, P-(NIPAM-co-AA) copolymer microgels appear to be more suitable for the preparation of “smart” emulsions, whose stability can be controlled by varying the temperature or acidity. This is because a change in the conformation of the particles significantly influences the interfacial diffusion coefficient with temperature. Additionally, their structure allows for a noticeable change in size in response to a change in pH, as we demonstrated, for example, in [45]. Meanwhile, IPN microgels, due to their unique architecture, allow us to obtain emulsions with high colloidal stability. As we established using computer modeling [40] when the thermoresponsive PNIPAM subnetwork collapses with the increasing temperature, the pH-responsive PAA subnetwork remains swollen and the whole particle preserves the three-dimensional shape. This provides the stability of the IPN microgel-stabilized emulsions in a wide range of temperatures.

## 4. Materials and Methods

### 4.1. Materials

N-isopropylacrylamide (NIPAM—monomer, Sigma-Aldrich, Munich, Germany, molecular weight (M) = 113.16 g/mol), N,N’-methylene-bisacrylamide (BIS—cross-linking agent, Sigma-Aldrich, Munich, Germany, M = 154.17 g/mol), ammonium persulfate (AMPS—initiators, Sigma-Aldrich, Munich, Germany, M = 154.17 g/mol), potassium persulfate (KPS—initiator, Sigma-Aldrich, Munich, Germany, M = 270.32 g/mol), sodium dodecylbenzenesulfonate (SDBS—surfactant, Sigma-Aldrich, Munich, Germany, M = 348.48 g/mol), tetramethylethylenediamine (TEMED, Sigma-Aldrich, Munich, Germany, M = 116.20 g/mol) andtetradecane (Sigma-Aldrich, Munich, Germany, M = 198.38 g/mol) were used as received. Acrylic acid (AA—monomer, Sigma-Aldrich, Munich, Germany, M = 72.06 g/mol) was purified by distillation. Water was purified using a Millipore Milli-Q system (Merck, Darmstadt, Germany).

### 4.2. PNIPAM Microgels and Co-Polymer Microgels Synthesis

Microgel particles of PNIPAM and its copolymer with acrylic acid (AA) were synthesized via free-radical thermo-induced precipitation polymerization in aqueous solutions, using BIS as a cross-linking agent. Ammonium persulfate (APS) was used as an initiator. A total of 0.05 g of SDBS surfactant was added per 100 mL of the reaction mixture for the preparation of P-(NIPAM-co-AA) microgels. Polymerization was carried out in a glass reactor equipped with a thermostatically controlled chamber and a magnetic stirrer, under a nitrogen atmosphere at a temperature of 80 °C, with constant stirring at a speed of 800 rpm for 24 h. The resulting microgels were washed to remove reagent residues by dialysis (dialysis tubes with molecular weight cut-off 20,000). The ratios of monomers, initiator and crosslinking agent used in the synthesis are provided in Table 3. The choice of ratios used is based on the observation that they lead to the formation of microgels with an optimal crosslinking density, allowing for a noticeable response to temperature changes. In the case of copolymer microgels, these ratios facilitate the production of particles in which the molar fraction of acrylic acid units is comparable to the fraction of the polyacrylic acid network within the IPN microgels. The content of acrylic acid units in P-(NIPAM-co-AA) microgels was found equal to 23–25%mol [54].

### 4.3. PNIPAM/PAA IPN Microgels Synthesis

IPN microgels were obtained by free-radical redox initiated polymerization of acrylic acid in the presence of cross-linking agent BIS within PNIPAM microgel matrices. The feasibility of this method for obtaining IPN microgels was demonstrated by Xia et al. [55]. It has been shown that using NIPAM as a monomer for the initial microgel formation and acrylic acid as a monomer for the second network significantly increases the concentration of AA within the volume of PNIPAM microgels. This increase is attributed to the formation of hydrogen bonds between these two types of monomer units [56]. As a result, PNIPAM-PAA IPN microgels were successfully synthesized. In this work, we implemented the same principle of IPN formation. Additionally, as we demonstrated earlier, it is important to control the synthesis time to ensure that the acrylic acid network does not grow beyond the microparticle matrix.

The synthesis procedure was the following: 350 µL acrylic acid, 0.075 g BIS were added to the mixture of 90 mL water and 15 mL 0.1 w.% solution of matrix microgels. The obtained mixture was stirred (800 rpm) for 30 min. After 2.5 mL water solution of AMPS (0.05 M) was added. Then, 37.5 µL TEMED was added in 15 min. Reaction time was 120 min. Polymerizations were carried out under a nitrogen flow at 23 °C. Resulting solutions were purified by dialysis (molecular weight cut-off 20,000) for 7 days. The content of acrylic acid units in PNIPAM-PAA IPN microgels was found equal to 29% mol [57].

### 4.4. DLS and SLS Measurements

Static and dynamic light scattering (SLS and DLS) measurements were conducted to determine the particle size of microgels at temperatures below (20 °C) and above (40 °C) the lower critical solution temperature (LCST) of PNIPAM, using a static/dynamic compact goniometer (DLS/SLS-5000, ALV, Langen, Germany). A HeNe laser with a power of 22 mW emitting a polarized light at *λ* = 632 Å was used as the incident beam. Distributions over decay time *τ* and hydrodynamic radius *R_h_* were obtained by means of a nonlinear regularized inverse Laplace transformation method (CONTIN) [58]. Radii of gyration *R*_g_ were calculated from the plots of the reciprocal excess intensity normalized on the relative contribution of *I*(*q*) vs. *q*^2^ using Guinier relation [59]:

(5) $$\ln\left(Kc/R_{\theta }\right)=\ln\left(\frac{1}{M_{w}\exp\left(-\frac{1}{3}R_{g}^{2}q^{2}\right)}+2A_{2}c\right)$$

where $K=4\pi^{2}{\left(dn/dc\right)}^{2}n_{o}^{2}/N_{o}\lambda^{4}$, *n*_0_ is the medium refractive index, *N*_0_ is the Avogadro number, *dn*/*dc* is the refractive index increment, *c* is concentration $R_{\theta }$ is the Rayleigh ratio at the angle *θ*, *M_w_* is molecular weight, and *A*_2_ is 2nd virial coefficient.

The studies of temperature dependences of average scattering intensity were carried out at a scattering angle of 60°.

### 4.5. Interfacial Tension (IFT) Measurements

The interfacial tension measurements were conducted using a Teclis Tensiometer Tracker S (Civrieux d’Azergues, France), equipped with a pendant drop module and a thermostatically controlled cell. The data analysis was conducted using the Drop Shape Analysis software WINDROP-XP 8.0 provided by the manufacturer. Measurements were conducted at the water-tetradecane interfaces using a rising drop mode. The experiment duration was set to 10,000 s, which is when significant changes in surface tension ceased for all samples. A small amount of 0.1 M NaOH was added to the aqueous solutions of P-(NIPAM-co-AA)/PNIPAM-PAA IPN microgels to adjust the pH to approximately 6, ensuring it remains higher than the pK of PAA when the acrylic acid groups are in their deprotonated state.

## Figures and Tables

**Table 1:** The results of microgels size and corresponding scattering intensity of microgel solutions measurements at temperatures below (20 °C) and above (40 °C) the lower critical solution temperature (LCST) of PNIPAM.

Temperature, °C	*R*_h_, nm	*R*_g_, nm	*R*_g_/*R*_h_	*I* * (kHz)
PNIPAM microgels				
20	333	167	0.5	1087 ± 43
40	119	88	0.7	8430 ± 298
P-(NIPAM-co-AA) microgels				
20	590	159	0.3	4121 ± 202
40	475	158	0.3	4350 ± 82
PNIPAM-PAA IPN microgel				
20	496	146	0.3	145 ± 8
40	418	142	0.3	164 ± 4

* at microgel concentration in solution *C* = 5·10^−5^ g/mL.

**Table 2:** Comparison between normalized interfacial diffusion coefficient *D^n^_interfacial_* at 20 °C and 40 °C.

MG Name	*D_interfacial_* *	
	20 °C	40 °C
P-(NIPAM-co-AA)	1.0	7.8
PNIPAM	2.7⋅10^5^	2.7⋅10^5^
PNIPAM-PAA IPN	3.3⋅10^5^	3.7⋅10^5^

* *D^n^_interfacial_* is normalized with *D_interfacial_* of P-(NIPAM-co-AA) microgels at 20 °C.

**Table 3:** The concentrations of monomers and the crosslinking agent during the synthesis of PNIPAM microgels and co-polymer microgels.

MG Type	NIPAM *	AA *	APS	BIS *
PNIPAM	1	0	0.01	0.02
P-(NIPAM-co-AA)	1.5	0.43	0.0125	0.03

* g per 100 mL of the reaction mixture.

## Data Availability

The original contributions presented in this study are included in the article. Further inquiries can be directed to the corresponding authors.
